# The Effects of *Saposhnikovia divaricata* Aqueous Extracts on the Inflammation and Intestinal Microflora in Allergic Rhinitis Mice

**DOI:** 10.1155/2022/1052359

**Published:** 2022-10-14

**Authors:** Yanchun Chen, Zhiling Chen, Gan Wang, Shiying Xu

**Affiliations:** Department of Otolaryngology, Hangzhou Hospital of Traditional Chinese Medicine, Hangzhou 310007, China

## Abstract

**Background:**

Allergic rhinitis (AR) is a type I allergic disease induced by IgE. Traditional Chinese medicine *Saposhnikovia divaricata* (Turcz.) Schischk (SD) has anti-inflammatory and antiallergic effects.

**Materials and Methods:**

AR model mice were constructed with ovalbumin (OVA) sensitization to observe the improving effect of SD treatment on AR by counting the number of sneezing and rubbing the nose, hematoxylin-eosin, periodic acid-Schiff, and toluidine blue stains. In addition, the allergy and inflammatory cytokines levels and inflammatory cells were observed by ELISA and Wright's-Giemsa stain. The protein levels of the TLR4/TRAF6/NF-*κ*B and IL-6/ROR-*γ*t/STAT3 pathways were measured by immunohistochemistry, quantitative real-time PCR, and western blot. The intestinal microflora abundance in mice was observed by 16S rDNA high-throughput sequencing.

**Results:**

SD treatment inhibited the sneezing and rubbing times of the nose, decreased the degree of a dense arrangement of cells and mucosal swelling and the number of goblet and mast cells of nasal lavage fluid, reduced the levels of IgE, histamine, Leukotriene B4, IL-4, IL-5, TNF-*α*, IL-6, and IL-17, the eosinophils, neutrophils, and lymphocytes number, the LR4, TRAF6, IL-6, ROR-*γ*t, and STAT3 mRNA levels, respectively, while, it increased the IL-2, IL-10, IFN-*γ*, and TGF-*β*1 proteins. SD treatment inhibited the NF-*κ*B, p-STAT3, TLR4, TRAF6, and p-I*κ*B*α*/I*κ*B*α* proteins. Besides, the effects of OVA and SD treatments were significantly correlated with the abundance of intestinal microflora. The abundances of *Cytophagales, Burkholderia, Alteromonadales, Lactococcus,* and *Clostridiaceae* were changed in SD treatment on AR mice.

**Conclusions:**

This study provides a possibility that the improvement effect of SD treatment on allergies and inflammation in AR mice may be related to the TLR4/TRAF6/NF-*κ*B and IL-6/ROR-*γ*t/STAT3 pathways and intestinal microflora modulation.

## 1. Introduction

Allergic rhinitis (AR) is an inflammatory disorder of nasal mucosa with a high prevalence that affects people of all ages [1]. AR is a type I allergic disease by IgE with nasal itching, paroxysmal sneezing, nasal hypersecretion, nasal congestion, and other symptoms [2]. AR has developed into a global public health problem. It is not a fatal disease, but it is the basis of many complications and can severely affect the quality of life without effective treatment [3]. Mast cells (MCs) have been considered key effector cells in allergic reactions, and the IgE on them causes an allergic reaction. When patients are exposed to allergens again, allergen-specific IgE will combine with the allergens, resulting in the rapid release of mediators such as histamine (HIS) causing nasal reactions such as sneezing, nasal discharge, and nasal itching [4]. In addition, the production of cytokines such as HIS, tumor necrosis factor-*α* (TNF-*α*), and leukotriene (LT) can promote the inflow of inflammatory cells and further aggravate AR [5]. Xu reported that inhibition of toll-like receptor 4 (TLR4) on improving the AR via NF-*κ*B and TNF receptor-associated factor 6 (TRAF6) played a key role in the TLR4-induced NF-*κ*B activation [6]. Moreover, Hua's team found that the native monocytes in AR patients were with the interleukin (IL)-6 up-regulated [7]. Piao's team reported that Saikosaponin A inhibited the IL-6/ROR-*γ*t/STAT3/IL-17 pathway to improve AR [8].

*Saposhnikovia divaricata* (SD), the dried root of *Saposhnikovia divaricata (*Turcz*.)* Schischk, is a traditional Chinese medicine with anti-inflammatory and antiallergic effects [9, 10]. Li has found SD protected arthritis rats and inhibited the level of inflammatory factors in serum [11]. Moreover, Wang's team observed the amelioration of SD treatment on FITC-induced allergic mice [12]. In particular, researchers studying the effects of polysaccharides of SD on AR races found it can inhibit the secretion of IgE, HA, and LTC4 [13]. In conclusion, SD has anti-inflammatory and antiallergic effects, but the biological mechanisms involved are not well defined. Therefore, we constructed AR model mice by ovalbumin (OVA) to observe the changes in behavioral and histopathological damage in mice after the action of SD and explored the biological mechanism by western blot (WB), immunohistochemistry (IHC), quantitative real-time PCR (qRT-PCR), and other techniques.

Besides, in recent decades, scientists found intestinal microflora was essential for host immune function, energy metabolism, and intestinal permeability [14]. Studies have demonstrated that the intestinal microflora could regulate type 2 immunity, regulate basophil hematopoiesis, and determine the trend of the host immune response [15]. What is more, a study has reported that *Clostridium butyricum* could promote the expression of IL-10 by antigen-specific B cells, leading to improved immune indicators in AR patients with the downregulation of IgE and Th2 [16]. Especially, scientists revealed that colon ROR*γ* Treg cells were induced by IL-6 which was related to intestinal microflora. Therefore, this study explored the correlation between the etiology of AR and intestinal microecology by 16s rDNA high-throughput sequencing to provide new targets for the prevention and treatment of AR.

## 2. Materials and Methods

### 2.1. Aqueous Extract Preparation

In short, the SD that was provided and identified by the pharmacy of the author's hospital was decocted twice with 10 times and 8 times volume water, respectively, for 1 h each time, and the decoction was filtered by No.3 filter paper; the aqueous extract was concentrated using a rotary evaporator at 60°C and was vacuum dried [11]. Each 1 g of the concentrated aqueous extract is equivalent to 20 g of the original drug.

## 3. Animals and Grouping

The male BALB/*c* mice (6 weeks) were purchased by Shanghai Lingchang Biotech Co., Ltd. They were housed in a standard specific pathogen free grade environment with a free diet and light dark alternation with 14 h light and 10 h dark. The 24 mice were divided into three groups, namely, the control group, the AR group, and the SD group. Ovalbumin (OVA) (95%, 257–264, Sigma, USA) was prepared with 0.4% aluminum hydroxide to a concentration of 0.2 mg/mL. On the 1^st^ day, the mice in AR and SD groups were injected with 0.5 mL OVA, in which 0.05 mL was injected subcutaneously in each of two points on the hind paws-plantar, two points on the groins, and two points on the back, and 0.2 mL by intraperitoneal injection; on the 7^th^, 14^th^, and 21^st^ day, the mice were intraperitoneally injected with 0.5 mL OVA. On the 22^nd^ day, the mice in the AR and SD groups were dosed daily intranasally with 20 *μ*L of 25 mg/mL OVA for 14 days to consolidate allergic reaction. At the same time, the mice in the SD group were administered 16 mg/kg of SD aqueous extracts by gavage for 14 days according to the conversion of animal dose to a human dose based on body surface area [17], and mice in other groups were dosed with an equal volume of saline by gavage.

## 4. Animal Behavioral Observations

On the day after the last gavage, the number of sneezing and rubbing of the noses of each mouse was observed individually for 15 min on the final day of gavage.

## 5. Sample Preparation

On the day after the last gavage, nasal lavage fluid (NLF) was collected for the enzyme-linked immunosorbent assay (ELISA) and cytometry assay. The way of collecting the NLF was as Cho's team described [18]; in brief, two consecutive volumes of 350 *µ*l of PBS through the catheter (24G IV) that was inserted into the pharyngeal opening were instilled, and the NLF was collected from the nostrils. Subsequently, blood was extracted from the mouse orbit, the supernatant of blood was collected by centrifugation, and the serum was stored at −80°C. After euthanasia, the murine nasal maxillae were removed and immersed for fixation in 10% neutral formalin solution for 24 h; then, the intact nasal cavities were left to be fixed in 4% paraformaldehyde for histopathological examination.

### 5.1. Staining Analysis of Tissue Sections

After dewaxing and dehydrating, paraffin sections (4 *μ*m) were stained by the stain kits of hematoxylin-eosin (H&E), periodic acid-Schiff (PAS), and toluidine blue (TB) stains according to the routine steps [19–21]. Moreover, the sections were dehydrated again, and then, they were mounted with neutral gum and examined by microscopy (ECLIPSE E100, Nikon, Japan). The sections by H&E stain were scored according to the degree of a dense arrangement of cells and the degree of mucosal swelling, the PAS stain was used to observe the number of goblet cells, and the TB stain was used to count the number of mast cells. In PAS stain, goblet cells are purplish-red, and nuclei are blue. In TB stains, the mast cells are dark purple.

### 5.2. Enzyme-Linked Immunosorbent Assay

The levels of immunoglobulin *E* (IgE), histamine (HIS), leukotriene B4 (LTB4), IL-2, 4, 5, 6, 10, 17, interferon-*γ* (IFN--*γ*), tumor necrosis factor-*α* (TNF-*α*), transforming growth factor-*β*1 (TGF-*β*1) in serum, or NLF were measured by ELISA according to instructions. The ELISA kits of IgE (MM-0056M1), HIS (MM-0548M1), LTB4 (MM-0041M1), IL-4 (MM-0165M1), IL-5 (MM-0164M1), IFN-*γ* (MM-0182M1), IL-6 (MM-0163M1), and IL-17 (MM-0170M1) were purchased from Jiangsu Meimian, Co., Ltd., China. Moreover, the IL-2 and TGF-*β*1 ELISA kits were provided by Shanghai Enzyme-linked Biotechnology Co., Ltd., China.

### 5.3. Cell Counting

After centrifugation, the precipitate of NLF was resuspended in 1 mL PBS (100 mM) supplemented with 1% bovine serum albumin (BSA) (G5001, Servicebio, China). Eosinophils, neutrophils, and lymphocytes were stained by Wright's-Giemsa stain (C0133, Beyotime, China) for cell counting [22]. The number of cells was counted by using a hemocytometer.

## 6. Immunohistochemistry (IHC)

After dewaxing and dehydrating, the tissue section (4 *μ*m) was subjected to citrate antigen retrieval solution for antigen retrieval, and endogenous peroxidase of it was blocked by H_2_O_2_. Moreover, 3% BSA was used to block the tissue section. Subsequently, the NF-*κ*B p65 (1 : 200, AF5006, Affinity, US) and p-STAT3 (1 : 200, AF3293, Affinity, US) antibodies were incubated with the sections separately at 4°C overnight. After incubation by the corresponding secondary antibody, tissue sections were sequentially stained by a DAB kit (G1212, Servicebio, China) and hematoxylin (G1340, Servicebio, China) [23]. Lastly, they were dehydrated and mounted. Microscopy was used for observation and analysis. The positive expression was brown-yellow, and the nuclei were blue.

### 6.1. Quantitative Real-Time PCR (qRT-PCR)

Generally speaking, after total RNA was extracted from mouse nasal mucosa by using the Trizol kit (B511311, Sangon Biotech, Shanghai, CHN), and reverse transcription was performed by using the HiFiScript cDNA Synthesis Kit (CW2569, CWBIO, Beijing, CHN). PCR was carried out under the following conditions by SYBR Premix Ex TaqII according to instructions, 95 °C for 10 min and 95 °C for 15 s, then at 60 °C for 60 s, performed 40 cycles [24]. The data were processed by the relative quantitative method (2^−ΔΔCt^). The primer information is shown in Table 1.

### 6.2. Western Blot (WB)

The RIPA lysate buffer (P0013D, Beyotime, CHN) was used to obtain the total proteins from mouse nasal mucosa, and a BCA kit (pc0020, Solarbio, CHN) was used to measure the concentration of total proteins. Then, protein denaturation was used by way of 100°C boiling water for 5 min, and the proteins were separated by electrophoresis. Later, the polyvinylidene fluoride membranes were used to transfer proteins in electroblotting, and then they were blocked by 5% nonfat milk. After washing by TBST and incubating with the TLR4 (1 : 2000, AF7017), TRAF6 (1 : 2000, AF5376), p-I*κ*B*α* (1 : 2000, AF2002), I*κ*B*α* (1 : 2000, AF5002), NF-*κ*B p65 (1 : 1500, AF5006), ROR-*γ*t (1 : 500, DF3196), p-STAT3 (1 : 2000, AF3293), STAT3 (1 : 2000, AF6294), and *β*-actin (1 : 10000, AF7018) antibodies at 4 °C overnight, the samples reacted with the corresponding secondary antibody for 2 h at 25 °C next day. Moreover, an ECL chemiluminescence kit (PE0010, Solarbio, Beijing, CHN) was used to color the protein band; subsequently, the membranes with the protein band were imaged with a chemiluminescence instrument (610020-9Q, Qin Xiang, Shanghai, CHN). The antibodies were all provided by Affinity Biosciences Ltd., Co, USA.

### 6.3. 16S rDNA High-Throughput Sequencing

After euthanasia, the caecum contents of 5 mice in each group were taken and handed over to Suzhou PANOMIX Biomedical Tech Co., Ltd. The total DNA of intestinal contents of mice was quantified by NanoDrop 2000, and the quality of DNA was detected by 1.2% agarose gel electrophoresis. The genome was amplified by PCR to obtain the v4-v5 region of the bacterial 16SrRNA gene. The Vazyme VAHTSTM DNA Clean Beads were added to the purification and recovery of amplification products. The recovered products of PCR amplification were quantified by the Quant-iT PicoGreen dsDNA assay kit, and the quantitative instrument was a microplate reader (BioTek, flx800). According to the sequencing quantity requirements of each sample, each sample shall be mixed according to the corresponding proportion. The sequencing library was prepared with the TruSeq Nano DNA LT Library Prep kit of Illumina company, and then high throughput sequencing was started. Sequence denoising or OTU clustering was performed according to QIIME2 dada2 or VSEARCH, and subsequently, QIIME2 (2019.4) was also used for species composition, alpha diversity, and beta diversity analysis. Based on KEGG (Kyoto Encyclopedia of Genes and Genomes, https://www.genome.jp/kegg/) and MetaCyc [25] (Metabolic Pathways From all Domains of Life, https://metacyc.org/) databases, the microbial metabolic function of samples was predicted to find out different pathways and obtain the species composition of specific pathways. The enrichment pathway analysis was carried out based on intestinal microflora that has significant differences in the abundance of groups.

### 6.4. Statistical Analysis

If the metrology data were normally distributed and conformed to the homogeneity of the variance test, it was analyzed by the Turkey test; if the data were in normal distribution but unequal variance, the data met the paired Student's *t*-test. All data were analyzed by SPSS 16.0. software and expressed as the mean ± standard deviation, and *P* < 0.05 means the difference was statistically significant.

## 7. Results

### 7.1. SD Treatment Improved the Disease Behavior and Histopathological Injury of AR Mice

**Figure 1:**
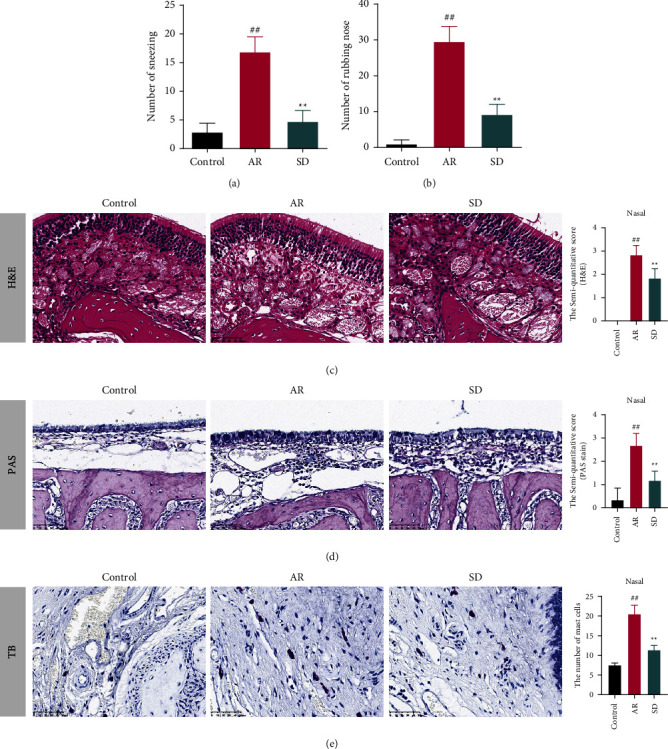
The effects of SD treatment on allergic rhinitis of AR mice. (×400) SD reduced the number of (a) sneezing and (b) rubbing noses in AR mice ((*n*) = 8). (c) The histopathological changes of the nose in AR mice were observed by H&E stain, which was scored according to the degree of a dense arrangement of cells and the degree of mucosal swelling. The score was proportional to the degree of injury ((*n*) = 6). (d) The number of goblet cells in the AR mice nose was observed by PAS stain, which was proportional to the semiquantitative score ((*n*) = 6). (e) The number of mast cells of the nose in AR mice was observed by TB stain. The mast cells are dark purple ((*n*) = 6). All error bars represent mean ± standard deviation. ^##^*P* < 0.01, compared to the control group; ^*∗∗*^*P* < 0.01, compared to the AR group. SD: *Saposhnikovia divaricata,* AR: allergic rhinitis, H&E: hematoxylin-eosin, PAS: periodic acid-Schiff, and TB: toluidine blue.

The observation of sneezing and rubbing of the nose behavior was performed to verify the improvement effect of SD on AR mice (Figures 1(a) and 1(b)). The numbers of sneezing and rubbing the nose in the AR group were significantly higher than those in the control group, while SD-treated mice reversed it (*P* < 0.01). The histopathological injury of nasal mucosal in AR mice was assessed by H&E, PAS, and TB stains. In the AR group compared to the control group, the degree of cellular disarrangement and mucosal swelling was much more severe (Figure 1(c)); the number of goblet cells and mast cells was significantly larger (Figures 1(d) and 1(e)) (*P* < 0.01). Moreover, SD treatment could markedly antagonize the above trend (*P* < 0.01).

### 7.2. SD Treatment on AR Mice Could Improve Inflammation and Allergy

**Figure 2:**
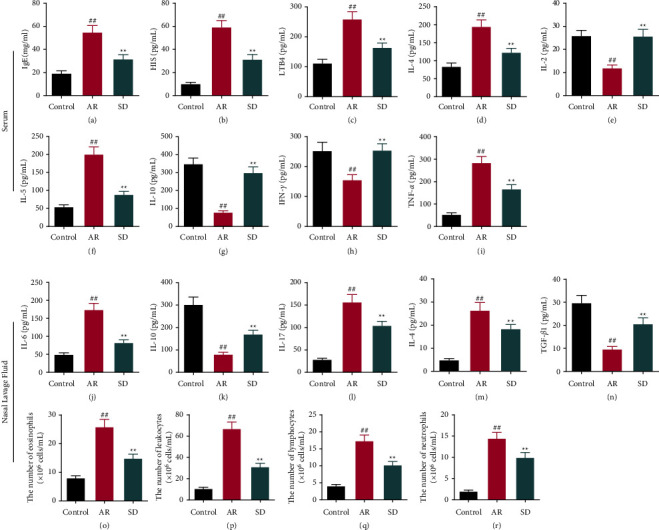
The effects of SD treatment on the levels of inflammation-related cytokines, allergy-related cytokines, and inflammatory cells in the serum and nasal lavage fluid of AR mice. ((a)–(n)) The levels of IgE, HIS, LTB4, IL-4, IL-2, IL-5, IL-10, IFN-*γ*, and TNF-*α* in the serum and the levels of IL-6, IL-10, IL-17, IL-4, and TGF-*β*1 in the nasal lavage fluids were measured by ELISA ((*n*) = 6). ((o)–(q)) The eosinophile granulocytes, leukocytes, lymphocytes, and neutrophils were stained by Wright's-Giemsa assay and counted ((*n*) = 8). All error bars represent mean ± standard deviation. ^##^*P* < 0.01, compared to the control group; ^*∗∗*^*P* < 0.01, compared to the AR group. SD: *Saposhnikovia divaricata*, AR: allergic rhinitis, IgE : immunoglobulin E, HIS: histamine, LTB4: leukotriene B4; IL: interleukins, IFN: interferon, TNF: tumor necrosis factor, TGF: transforming growth factor, and ELISA: enzyme-linked immunosorbent assay.

The levels of cytokines related to inflammation and allergy in the serum were measured by ELISA (Figures 2(a)–2(i)). The levels of IgE, HIS, LTB4, IL-4, IL-2, IL-5, and TNF-*α* in the AR group were significantly higher than those in the control group, and SD treatment on AR mice could inhibit those levels notably (*P* < 0.01). Moreover, the number of IL-2, IL-10, and IFN-*γ* was much lower in the AR group compared to the SD group, and the SD treatment could increase those levels markedly (*P* < 0.01).

In the NLF, the levels of IL-6, IL-17, and IL-4 were inhibited notably with SD treatment on AR mice (*P* < 0.01) (Figures 2(j), 2(l) and 2(m)), while the IL-10 and TGF-*β*1 levels were raised (*P* < 0.01) (Figures 2(k)and 2(n)). The numbers of eosinophils, leukocytes, lymphocytes, and neutrophils were significantly up-regulated in the AR group compared to the control group, and they were markedly down-regulated after SD treatment (*P* < 0.01) (Figures 2(o)–2(r)).

### 7.3. SD Treatment Regulated the TLR4/TRAF6/NF-Κb*κ* and IL-6/ROR-*γ*t/STAT3 Pathways in the Nasal Mucosa of AR Mice

**Figure 3:**
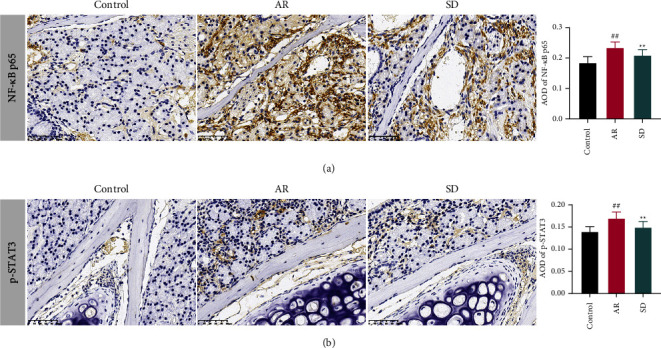
The effects of SD treatment on the levels NF-*κ*B p65 and p-STAT3 in the nose of AR mice ((*n*) = 6, ×400). Immunohistochemistry was used to detect (a) NF-*κ*B p65 and (b) p-STAT3 protein levels. The AOD was used as an index to evaluate protein levels. All error bars represent the mean ± standard deviation. ^##^*P* < 0.01, compared to the control group; ^*∗∗*^*P* < 0.01, compared to the AR group. SD: Saposhnikovia divaricata, AR: allergic rhinitis, AOD: average optical density, NF-*κ*B: nuclear factor kappa-B, and p-STAT3: phospho-signal transducer and activator of transcription 3.

The expression levels of NF-*κ*B p65 and p-STAT3 were measured by IHC. The average optical density of NF-*κ*B p65 and p-STAT3 was both markedly higher compared to the control group, and in the SD group, the average optical density of NF-*κ*B p65 and p-STAT3 was significantly reduced (*P* < 0.01) (Figures 3(a) and 3(b)).

**Figure 4:**
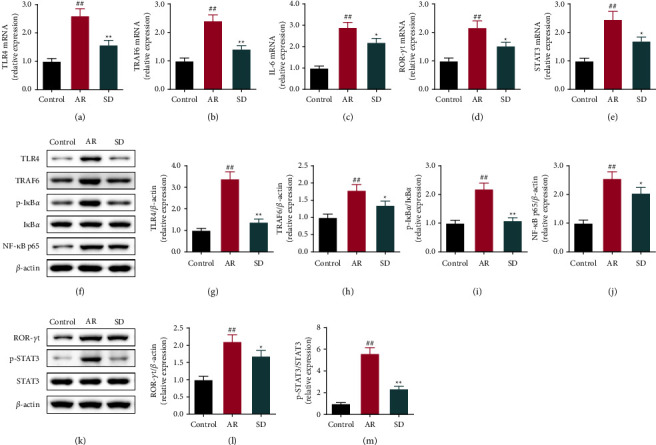
The effects of SD treatment on the levels of the TLR4/TRAF6/NF-*κ*B and IL-6/ROR-*γ*t/STAT3 pathways' related genes and proteins in the nasal mucosa of AR mice ((n) = 6). (A-E) The levels of TLR4, TRAF6, IL-6, ROR-*γ*t, and STAT3 mRNA were measured by quantitative real-time PCR. (F-M) The levels of TLR4, TRAF6, p-I*κ*B*α*/I*κ*B*α*, NF-*κ*B, ROR-*γ*t, and p-STAT3/STAT3 proteins were measured by using the western blot. All error bars represent the mean ± standard deviation. ^##^*P* < 0.01, compared to the control group; ^*∗*^*P* < 0.05, ^*∗∗*^*P* < 0.01, compared to the AR group. SD: Saposhnikovia divaricata, AR: allergic rhinitis, TLR4: toll-like receptors 4, TRAF6: TNF receptor-associated factor 6, IL-6: interleukins-6, I*κ*B*α*: inhibitor of NF-*κ*b, ROR-*γ*t: retinoid-related orphan nuclear receptor *γ*t, and STAT3: signal transducer and activator of transcription 3.

The mRNA levels of TLR4, TRAF6, IL-6, ROR-*γ*t, and STAT3 mRNA were measured by qRT-PCR (Figures 4(a)–4(e)). They were up-regulated in the AR group compared to the control group, while they were markedly reduced in the SD group than those in the AR group (*P* < 0.01, *P* < 0.05). The degrees of TLR4, TRAF6, p-I*κ*B*α*/I*κ*B*α*, and NF-*κ*B proteins were assessed by western blot (Figures 4(f)–4(m)). Moreover, all of the protein degrees were increased significantly in the AR group than those in the control group (*P* < 0.01). In the SD group, they are all decreased compared to the AR group (*P* < 0.01, *P* < 0.05).

### 7.4. SD Improved the OVA-Induced AR by the Intestinal Microflora Modulation

**Figure 5:**
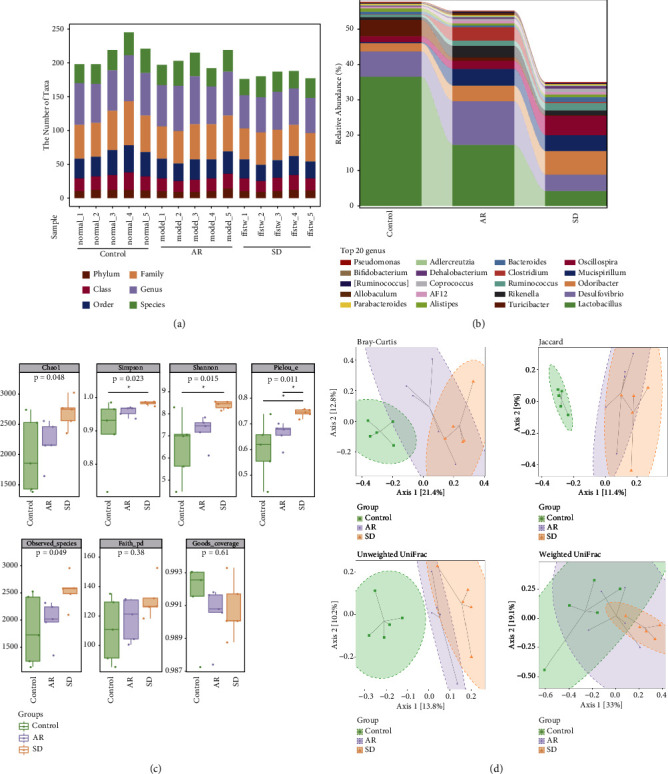
The effects of SD treatment on the intestinal microbial diversity of AR mice ((*n*) = 5). (a) The statistics of microbial taxa at phylum, class, order, family, genus, and species levels. There was large species abundance at the family and genus level. (b) The relative species abundance histograms on the genus. (c) The boxplots of the alpha diversity index. The Chao1 and observed species indexes are used to analyze the abundance, Shannon and Simpson are used to analyze diversity, the faith's PD index is used to characterize evolution-based diversity, Pielou's evenness index is used to characterize evenness, and Good's coverage index represents coverage. (d) The beta diversity was analyzed by PCoA that was based on the Bray–Curtis, Jaccard, unweighted UniFrac, and weighted UniFrac. All error bars represent the mean ± standard deviation. SD: *Saposhnikovia divaricata*, AR: allergic rhinitis, and PCoA: principal co-ordinates analysis.

The effects of SD on the community diversity of intestinal microbial in AR mice were tested by 16S rDNA high-throughput sequencing. After processing the abundance table of ASV/OTU by the qiime feature-table rarefy function in QIIME2, as shown in Figure 5(a), the number of units varies at each level (phylum, class, order, family, genus, and species) in different groups was counted. Moreover, in the genus level, the top 20 shown in Figure 5(b) are *Pseudomonas, Bifidobacterium, Ruminococcus, Allobaculum, Parabacteroide*s, and so on. The alpha diversity analysis of intestinal flora by different indexes is shown in Figure 5(c). In the SD group compared to the AR group, the alpha diversity of Pielou's evenness has a significant difference (*P* < 0.05). In Shannon and Simpson indexes, there are also significant differences in the SD group compared with the control group (*P* < 0.05). According to the beta diversity analysis of Jaccard and unweighted uniFrac based on PCoA, there were differences between the control group, the AR group, and the SD group (Figure 5(d)).

### 7.5. Analysis of Species Differences of the Intestinal Microflora among Different Groups

**Figure 6:**
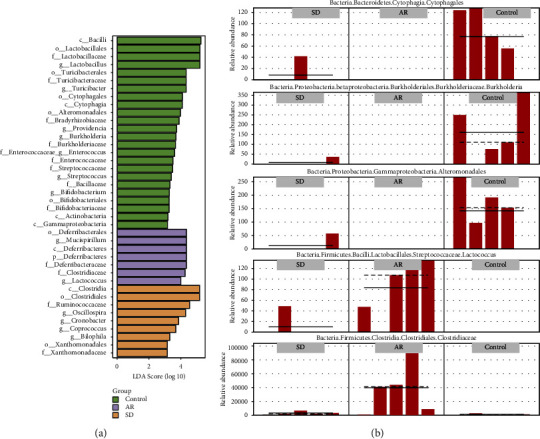
The species difference analysis of intestinal microflora in the AR mice with/without SD treated ((*n*) = 5). (a) The LDA effect size score of species with significant differences between the groups. The length is proportional to the significance of the difference, and the color indicates the group in which the species are with the highest abundance. (b) The relative abundance distribution of representative marker microorganisms in different groups. Solid and dashed lines indicate relative abundance mean and median values. SD: *Saposhnikovia divaricata*, and AR: allergic rhinitis.

**Figure 7:**
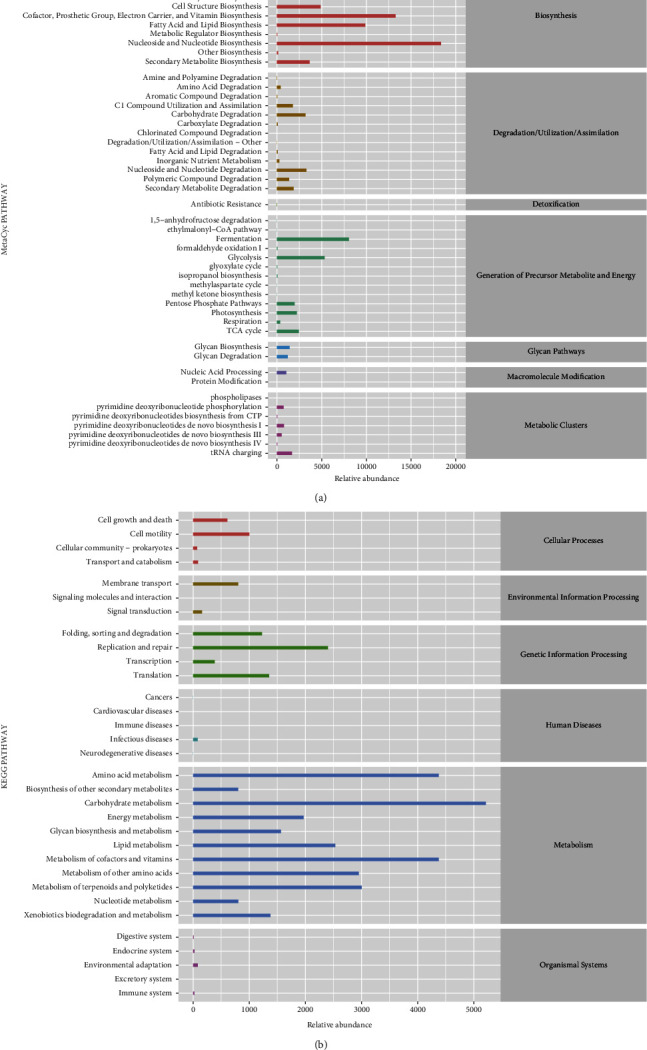
The species pathway enrichment analysis of intestinal microflora in the allergic rhinitis mice with/without SD treated ((*n*) = 5). (a, b) The predicted MetaCyc and KEGG pathway enrichment of marker microorganisms. This shows the average abundance across all samples. SD : *Saposhnikovia divaricata*, AR: allergic rhinitis, MetaCyc: metabolic pathways from all domains of life, and KEGG : Kyoto Encyclopedia of Genes and Genomes.

**Figure 8:**
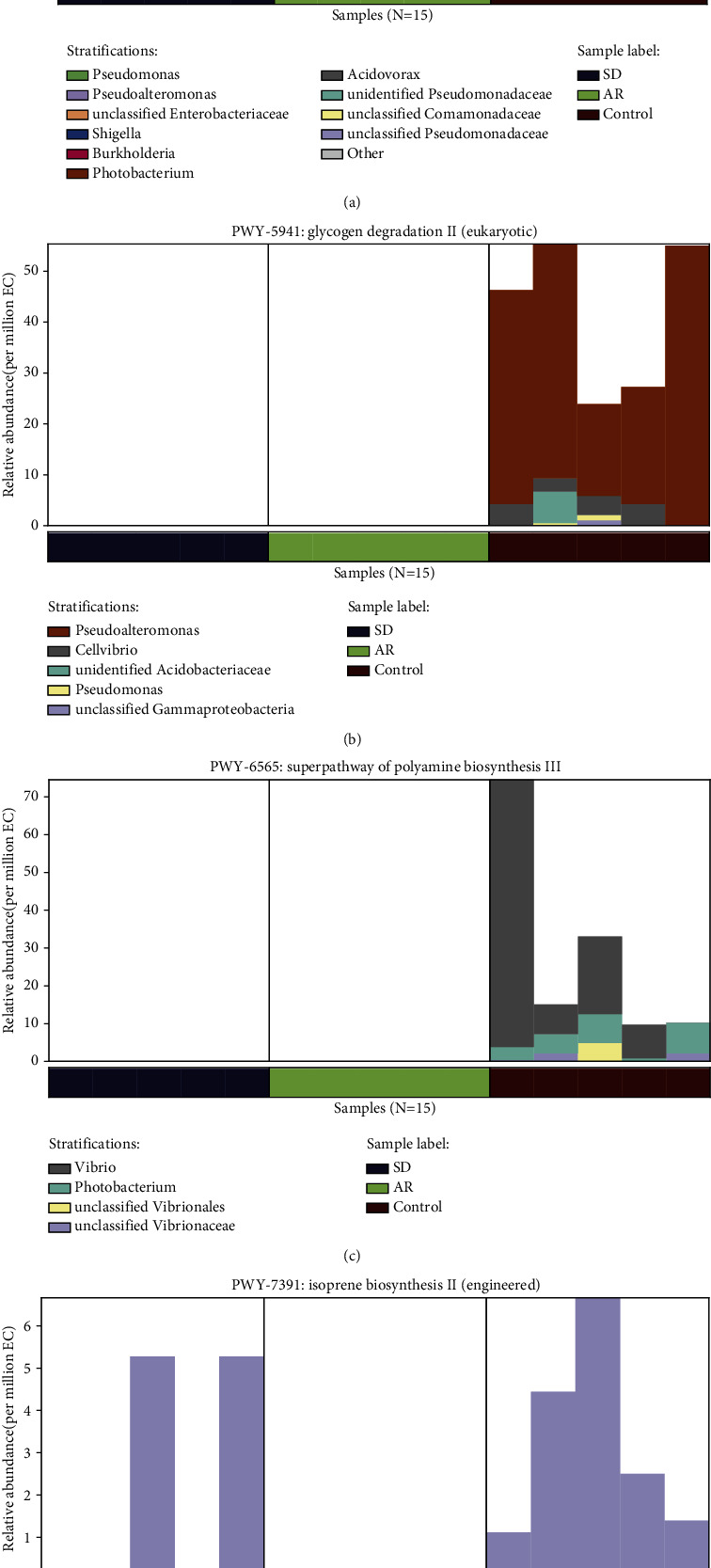
The species composition analysis of intestinal microflora in the AR mice with/without SD treated with significantly different pathways. Colors inside each group correspond to the bacterium, and the order indicates relative abundance. (a) In the L-arginine degradation II (AST) pathway, there are *Pseudomonas, Pseudoalteromonas, unclassified Enterobacteriaceae, Shigella, Burkholderia, Photobacterium, Acidovorax, unclassified Pseudomonadaceae, unclassified Comamonadaceae, unclassified Pseudomonadaceae*, and others. (b) In the glycogen degradation II (eukaryotic) pathway, there are *Pseudoalteromonas, Cellvibrio, unidentified Acidobacteriaceae, Pseudomonas*, and *unclassified Gammaproteobacteria*. (c) In the superpathway of polyamine biosynthesis III, there are *Vibrio, Photobacterium, unclassified Vibrionales,* and *unclassified Vibrionaceae*. (d) In the isoprene biosynthesis II (engineered) pathway, it is *Corynebacterium.* SD : *Saposhnikovia divaricata* and AR: allergic rhinitis.

The marker species were analyzed by LDA effect size analysis (Figure 6(a)). *c*_*Bacilli, o_Lactobacillales*, and *f*_*Lactobacillaceae* were the top 3 with the highest abundance in the control group; *o*_*Deferribacterales, g_Mucispirillum,* and *c*_*Deferribacteres* were the top 3 with the highest abundance in the AR group; *c_Clostridia, o_Clostridiales*, and *f*_*Ruminococcaceae* were the top 3 with the highest abundance in the SD group. The distribution of the relative abundance of marker species (*Cytophagales, Burkholderia, Alteromonadales, Lactococcus,* and *Clostridiaceae*) in different groups is shown in Figure 6(b), and it showed that the abundance of these markers was significantly changed in the AR mice after SD treatment. The enrichment analysis on MetaCyc and KEGG pathways was counted for marker species to find out the metabolic pathways with a potential high correlation (Figures 7(a) and 7(b)). The enrichment pathways were the L-arginine degradation II, glycogen degradation II, superpathway of polyamine biosynthesis III, and isoprene biosynthesis II pathways (Figures 8(a)–8(d)). In the L-arginine degradation II (AST) pathway, *Pseudomonas* is a common bacterium of the three groups, and its relative abundance ranks first (Figure 8(a)). In the glycogen degradation II (eukaryotic) pathway and the superpathway of polyamine biosynthesis III, *Pseudoalteromonas* and *Vibrio* had the largest abundance in the control group (Figures8(b), (c)). Interestingly, in the isoprene biosynthesis II (engineered) pathway, there is only *Corynebacterium* in the control and SD groups (Figure 8d).

## 8. Discussion

Traditional Chinese medicine has a definite and stable role in the prevention and treatment of AR with the advantages of overall regulation, multidirectional and multitarget [26]. Lenon studied the effect of a Chinese herbal formula (RCM-101) on rat peritoneal mast cells that inhibited HIS, LTB4, and prostaglandin E2 [27]. Yu-Ping-Feng-San (YPFS), a Chinese herbal medicine formula, was proved beneficial for treating adult AR [28], and Chen found that the potential therapeutic effect of YPFS on AR mice may be via *T* helper 1 and 2 [29]. SD was a key medicine of YPFS, which has been reported to be inhibiting the immunoregulatory mediators, TNF-*α*, IFN-*γ*, and IL-1*β*, in U937 cells [30]. Therefore, this study explored the effect and biological mechanism of SD on AR mice and proved that SD could improve disease behavior and histopathological injury.

Especially, SD reduced the number of mast cells in the nasal mucosa and the levels of inflammatory factors in the serum and NLF of AR mice in this study. Mast cells are the key effector cells of anaphylaxis and play an important role in the regulation of immune response [31]. Researchers found that the high level of IgE was related to the accumulation of mast cells [32], and IgE was a key receptor of mast cell degranulation, that is, the basis of type I hypersensitivity and inflammation [33]. The multiple inflammatory mediators of MCS degranulation and metabolites generated by membrane phospholipid metabolism are involved in the nasal allergic response to AR, such as HIS and LT [34], and they are strongly associated with the pathological manifestations of sneezing, scratching the nose, and tissue edema in AR [35]. LTB4 is key for the activation of mast cells and an attractant for their progenitor, which initiates inflammatory responses and promotes the release of inflammatory cytokines [36, 37]. What is more, a study reported that in an HDM-induced eosinophilic airway-inflammation mouse model, inhibition of LTB4 receptors restrained the number of eosinophils and the levels of IL-4, 5, and 13 to relieve pulmonary inflammation [38]. In short, this study observed that SD treatment on AR mice can improve sneezing and rubbing of the nose, reduce nasal mucosa swelling, and inhibit the levels of inflammatory cells and factors, which may be related to the reduction in IgE, HIS, and LTB4.

In addition, we studied the expression degrees of the TLR4/TRAF6/NF-*κ*B and IL-6/ROR-*γ*t/STAT3 pathways by IHC, qRT-PCR, and WB. A study on patients with persistent allergic rhinitis found that the TLR4 mRNA level was significantly higher compared to the control group [39]. The inhibition of TLR4/TRAF6/NF-*κ*B signaling improved AR, and Wang reported the alleviation of miR-146a on the inflammation in AR mice by inhibiting the TLR4/TRAF6/NF-*κ*B pathway [40]. Besides, Piao's team reported that Saikosaponin A attenuated the AR via regulating the levels of the IL-6/ROR-*γ*t/STAT3/IL-17/NF-*κ*B pathway. It has been reported that MCs could amplify the immune response of T cells in the presence of TLR4 which is required for the induction of immune responses by MCs [41]. This study observed that SD restrained the protein levels of the TLR4/TRAF6/NF-*κ*B and IL-6/ROR-*γ*t/STAT3 pathways, which may be the one of reasons that SD improved AR.

In recent decades, studies have found that the dysbiosis of intestinal microflora was closely related to the development of allergic diseases [42]. Germ-free mice have a significantly lower density of mast cells in the small intestine than normal mice, and the migration of mast cells from the blood to the intestine is also associated with intestinal microflora [43]. Studies have demonstrated that the gut microbiota could regulate type 2 immunity, regulate basophil hematopoiesis, and determine the trend of the host immune response [15]. Besides, a study has reported that *Clostridium butyricum* could promote the expression of IL-10 by antigen-specific B cells, leading to improved immune indicators in AR patients with the downregulation of IgE and Th2 [16]. In this study, we found dysbiosis in the intestinal microflora of AR mice, and after SD treatment, the intestinal microflora abundances were changed. The abundances of *Bacteroidetes*, *Proteobacteria*, and *Firmicutes* in AR mice were significantly different from those in control mice. A study reported that *Bacteroidetes* was related to the improvement effect of the compound small peptide of Chinese medicine on intestinal immunity in mice [44]. Moreover, *Burkholderia*, which belonged to *Proteobacteria*, in intestinal contents of fish was increased by the growth-promoting traditional Chinese medicine treatment [45]. Kim's team reported that the abundance of *Firmicutes* was decreased when atopic dermatitis in mice was improved [46]. Similarly, this analysis of intestinal microflora in this study reported that those microflorae may play a role in the improvement of AR, which deserves further study. The interaction and biological mechanism between AR and intestinal microflora have the value of further exploration. Of course, the analysis results of the intestinal microflora are all predictions. If the sample number can be increased, a more accurate analysis of the intestinal microflora can be carried out. In summary, this study provides experimental evidence for the future development of SD intervention in AR and provides new ideas and the basis for the development of new drugs against allergic rhinitis in traditional Chinese medicine.

## 9. Conclusions

To summarize, this paper proved the improving effect of SD on behavior and histopathology of AR mice, and SD treatment inhibited the levels of proinflammatory and immune mediators (IgE, HIS, LTB4, IL-4, 5, 6, 17, TNF-*α*, and TGF-*β*1) and raised the levels of anti-inflammatory mediators (IL-2, 10, and IFN-*γ*) in OVA-induced AR mice. Moreover, SD treatment regulated the levels of NF-*κ*B, TLR4, TRAF6, p-I*κ*B*α*/I*κ*B*α*, ROR-*γ*t, and p-STAT3/STAT3 proteins and intestinal microflora in AR mice. This study suggested that the improvement effect of AR by SD treatment is related to the TLR4/TRAF6/NF-*κ*B, IL-6/ROR-*γ*t/STAT3 pathways and intestinal microflora modulation. The results of this study provide a new idea and the basis for traditional Chinese medicine on AR treatment.

## Figures and Tables

**Table 1:** Primer sequence.

Gene	Forward primer (5′-3′)	Reverse primer (5′-3′)
Mouse TLR4	AGCTCCTGACCTTGGTCTTG	CGCAGGGGAACTCAATGAGG
Mouse TRAF6	TACGATGTGGAGTTTGACCCA	CACTGCTTCCCGTAAAGCCAT
Mouse IL-6	GGCGGATCGGATGTTGTGAT	GGACCCCAGACAATCGGTTG
Mouse ROR-*γ*t	TCCACTACGGGGTTATCACCT	AGTAGGCCACATTACACTGCT
Mouse STAT3	CACCTTGGATTGAGAGTCAAGAC	AGGAATCGGCTATATTGCTGGT
Mouse GAPDH	AATGGATTTGGACGCATTGGT	TTTGCACTGGTACGTGTTGAT

## Data Availability

The data used to support the findings of this study are available from the corresponding author upon request.
